# Nutrient Limitation in Northern Gulf of Mexico (NGOM): Phytoplankton Communities and Photosynthesis Respond to Nutrient Pulse

**DOI:** 10.1371/journal.pone.0088732

**Published:** 2014-02-14

**Authors:** Yan Zhao, Antonietta Quigg

**Affiliations:** 1 Department of Oceanography, Texas A&M University, College Station, Texas, United States of America; 2 Department of Marine Biology, Texas A&M University at Galveston, Galveston, Texas, United States of America; Mount Allison University, Canada

## Abstract

Although the Mississippi-Atchafalaya River system exports large amounts of nutrients to the Northern Gulf of Mexico annually, nutrient limitation of primary productivity still occurs offshore, acting as one of the major factors controlling local phytoplankton biomass and community structure. Bioassays were conducted for 48 hrs at two stations adjacent to the river plumes in April and August 2012. High Performance of Liquid Chromatography (HPLC) combined with ChemTax and a Fluorescence Induction and Relaxation (FIRe) system were combined to observe changes in the phytoplankton community structure and photosynthetic activity. Major fluorescence parameters (F_o_, F_v_/F_m_) performed well to reveal the stimulating effect of the treatments with nitrogen (N-nitrate) and with nitrogen plus phosphate (+NP_i_). HPLC/ChemTax results showed that phytoplankton community structure shifted with nitrate addition: we observed an increase in the proportion of diatoms and prasinophytes and a decrease in cyanobacteria and prymnesiophytes. These findings are consistent with predictions from trait-based analysis which predict that phytoplankton groups with high maximum growth rates (*μ_max_*) and high nutrient uptake rates (*V_max_*) readily take advantage of the addition of limiting nutrients. Changes in phytoplankton community structure, if persistent, could trigger changes of particular organic matter fluxes and alter the micro-food web cycles and bottom oxygen consumption.

## Introduction

Liebig' law of minimum first claimed that plant growth is not determined by the total amount of a resource, but instead limited by the scarcest resource (Liebig 1840 in [1]). Microalgal resource competition follows Liebig's law. Tilman et al. [2] resource ratio theory set up the basis for understanding use/competition for nutrient concentrations or ratios and phytoplankton community structure. Grover [3] established the variable-internal-stores model to offset the drawback of the applicability of Tilman's theory in non-steady states (eg. periodic or non-periodic nutrient pulses). The intermediate disturbance hypothesis emphasized that periods of nutrient pulse could also control the variability of phytoplankton community structure [4]. All these theories have been tested in laboratory and natural aquatic systems (mostly freshwaters) (e. g. [4], [5], [6]). Complex nutrient conditions in coastal environments lead to corresponding variability of phytoplankton community structure. The influence of fluctuating nutrient conditions on primary production, zooplankton grazing, particulate organic material cycling, and bottom oxygen consumption, are an important part of research in the Northern Gulf of Mexico (NGOM) [7]. It has been suggested that spring phytoplankton blooms are the initial step in the scenario of the development of annually bottom hypoxia [8].

Nutrient fluctuations in the NGOM are quite significant due to the large inputs of the Mississippi Atchafalaya River system, one of the ten largest rivers in the world [9]. In NGOM, nitrogen (N) limitation and phosphorus (P) limitation can both happen in different locations but during the same time frame [9], [10], [11]. As a result of this nutrient loading, dissolved inorganic nitrogen (DIN), inorganic phosphorus (P_i_), and silicate (Si) in the Mississippi River have increased to Redfield levels while DIN:P_i_ ratios in the NGOM exceed Redfield levels, particularly after high flows [12], [13]. Changing ratios of N:P:Si over the last 50 years imply that the limiting nutrient for primary production may also have changed [7], but this requires investigation. Research on nutrient limitation in this region has been conducted by direct nutrient measurements (e.g. [14]), resource limitation assays (RLAs) (e.g. [10], [11], [15]) and/or measurements of distinctive indicators (like enzymes, amino acids, proteins) (e.g. [16], [17]). With the development of fluorescence technology to measure phytoplankton biomass and physiology, this method has also been applied to the studies of nutrient limitation especially in combination with RLAs, also called nutrient addition assays (e.g. [15]).

RLAs have been suggested to be the better diagnostic tool for nutrient limitation than the direct measurement of nutrient concentrations and/or ratios [11]. RLAs have been done in NGOM. (e.g. [10], [11], [14], [15], [18]). However, all these studies focused on evaluating the nutrient status and the changes of phytoplankton biomass (or production) but not the community structure. The study of Lugus et al. [19] performed in the Baltic Sea showed phytoplankton community shifts occured after the addition of limiting nutrients in RLAs. In fact, there are certain patterns of phytoplankton responding to ambient nutrient stimulations. Litchman et al. [20] applied trait-based approaches in terrestrial ecology to the research of phytoplankton nutrient competition by means of proposing several nutrient-dependent functional traits which not only are species-specific, but also nutrient-specific. Trait-based ecology in phytoplankton communities has been widely shown in laboratory experiments but seldom in natural environments [21], [22].

Based on nutrient competition theory and trait-based ecology, phytoplankton community shifts may happen when nutrient conditions change. The object of this study was to investigate the effect of nutrient pluses on the phytoplankton community structure and physiology in NGOM in April and August 2012. In our research, we focused on the short-term (48 hr) response of phytoplankton communities under ambient conditions to changes only by the addition of nutrients, including nitrogen (as nitrate), organic and inorganic phosphate as well as a ‘bottom’ water sample (see below for definition). High Performance Liquid Chromatography (HPLC) combined with ChemTax was used in parallel with a Fluorescence Induction and Relaxation (FIRe) system to examine photosynthetic activities of the changing community, which is the first time this approach has been applied in NGOM studies. While previous studies have investigated the effect of nutrient pluses on phytoplankton biomass in NGOM (e.g. [10]–[18]), the shift in phytoplankton community structure in response to the pulse has not been examined.

## Materials and Methods

### Ethics Statement

All the field work involved in this study was approved by the National Oceanic and Atmospheric Administration. Our research area does not include privately owned or protected areas and protected animals. No animal husbandry, experimentation, and care/welfare were involved in our study.

### Sampling

**Figure 1:**
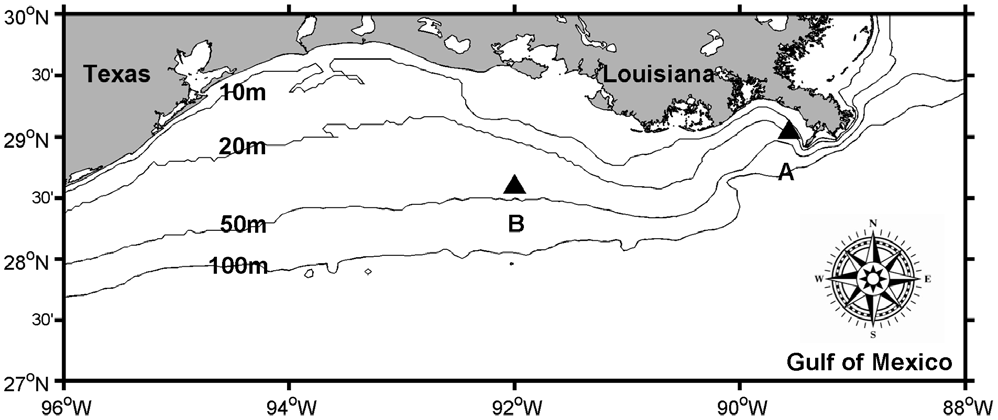
Study area and bathymetry for the mechanisms controlling hypoxia program in the northern Gulf of Mexico. The 10, 20, 50 and 100

**Figure 2:**
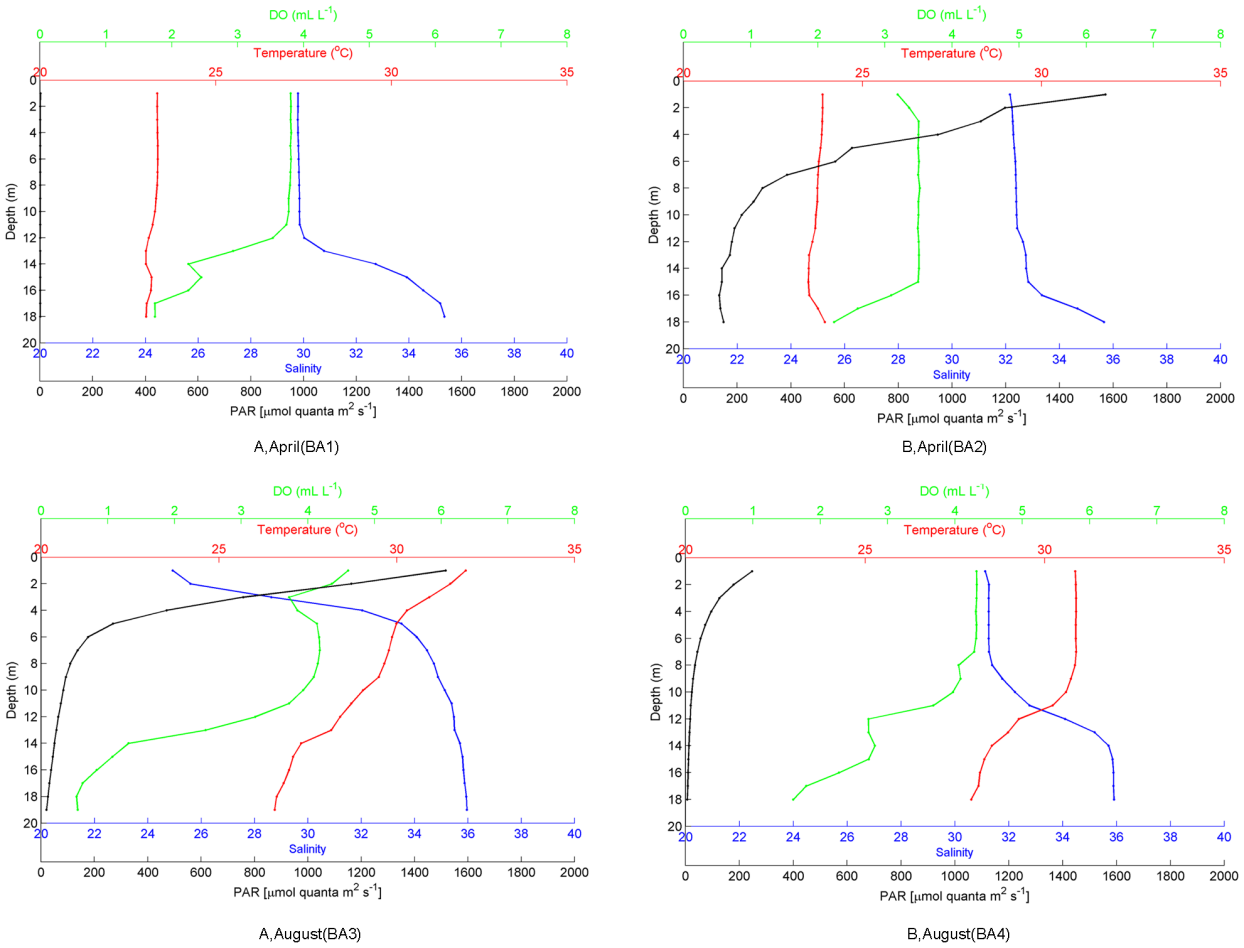
Hydrographic features of the water column immediately prior to the start of each bioassay (BA). Water column profiles were measured with calibrated sensors attached to the CTD rosette. At station A and station B in April (BA1 and BA2) and August (BA3 and BA4). The lack of PAR in BA1 was because the bioassay was started at night. The low PAR in BA4 was due to extensive cloud cover.

Four bioassays were conducted during cruises as part of the project ‘Mechanisms Controlling Hypoxia’ aboard the R/V *Pelican* in April and August 2012. The two sampling stations (A and B, located at 29.04°N, 89.56°W and 28.59°N, 92.00°W respectively) on the Louisiana Shelf are shown in Fig. 1. Surface water (0.5–2 m) was collected for in-situ bioassays (BA) using a CTD rosette with twelve 5 L Niskin bottles. Hydrographic parameters (temperature, salinity, PAR, and dissolved oxygen (DO)) were measured using shipboard calibrated sensors attached to the CTD rosette. Water column profiles immediately prior to sample collections are shown in Fig. 2. These are representative of the profiles measured (n≥12) as we remained at each station for no less than 24 hours and measured profiles at least every 2 hours. The four bioassays referred to BA1, BA2, BA3 and BA4 correspond to those performed in April at station A and B and then in August at station A and B respectively.

### Bioassays

The bioassays were performed essentially following the procedure of Fisher et al. [23]. In this study, the concentrations of nutrients added to bioassays were based on previous work performed by [15] and [10] in NGOM. Treatments, performed in triplicate 1 L bottles included control (no additions), +N (30 µmol L^−1^ NO_3_), +P_i_ (2 µmol L^−1^ PO_4_), +organic phosphorus (OP) (2 µmol L^−1^ D Glucose-6-phosphate), +NP_i_ (30 µmol L^−1^ NO_3_, 2 µmol L^−1^ PO_4_). We added two treatments to test the effect of grazers (NG) and ‘bottom’ water. Grazers were removed by filtering seawater though a 118 µm sieve before starting the bioassays (Michael Dagg, pers. comm.). No nutrients were added to the grazing treatments.

The ‘bottom+surface (SB) water’ treatment involved collecting waters from between ∼15 to 18 m along the 20 m isobath at the same time as the surface water using the CTD rosette. This was pre-filtered using a 0.2 µm cellulose ester filter (Millipore). The treatment consisted of 90% surface water +10% pre-filtered bottom water (no nutrients added). The aim of this treatment was to determine if the bottom water in the NGOM could stimulate the phytoplankton on the surface. Given the water column can be well stratified in the summer, previous observations have shown that nutrients accumulate under the pycnocline. During mixing events (e.g., hurricanes), these will be introduced quickly to the surface and may alleviate nutrient stress. This approach of examining bottom water nutrients on surface productivity has been applied elsewhere, such as Gulf of Aqaba and Qatar peninsula in Arabian Sea [24], [25].

The above seven treatments were incubated on deck in acid-washed polyethylene bottles for 48 hours in incubators with in-situ surface water continuously flowing through to maintain ambient temperatures. By using shade cloth, samples in the incubator received approximately 50% of ambient light. Samples were exposed to the natural light: dark photoperiod of 12 h: 12 h in April and 14 h: 10 h in August. Samples taken at the end of the incubation period will be referred to by their treatment, for example, control, +N, +P_i_ etc.

### Phytoplankton fluorescence

The Fluorescence Induction and Relaxation (FIRe) System (FIRe fluorometer, Satlantic Instruments S/N 2) was used to measure the photosynthetic parameters of the phytoplankton in the bioassays. Every 24 hours (that is, at 0, 24 and 48 hours), 3 ml water samples were taken out and stored in darkness for 30 minutes before measurements. Fluorescence from filtered seawater (0.45 µm) collected from the corresponding treatments was subtracted from the F_o_ and F_m_ values of samples to correct for the influence of background fluorescence [26]. In this study, we use only information collected from the single turnover (ST) component of the transient according to Kolber et al. [27] and Kromkamp and Forster [28], including the minimum fluorescence (F_o_), the photosynthetic efficiency of PS II (F_v_/F_m_), the functional absorption cross-section for PSII (σ_PSII_; Å^2^ quanta^−1^), the minimum turnover time of electron transfer between reaction centers (τ_PSII_; sec) and the connectivity factor (*p*) for the degree of departure from a simple exponential fluorescence rise (*p* = 0) towards a sigmoidal fluorescence rise during the FIRe trace (*p* approaches 1). The four parameters were sensitive to nutrient or light limitation, which were taken as physiological markers for nutrient limitation in many studies [15], [29], [30]. The light curves for all the samples were measured at different gain settings to ensure best signal to noise ratios. Gains were normalized in the calculations to account for these differences.

### Nutrients and pigment analysis

Before starting the bioassays, nutrient and chlorophyll (chl) *a* samples were taken to quantify the background nutrient concentrations and phytoplankton biomass. For nutrients, 20 mL water samples were filtered thought pre-rinsed 0.45 µm cellulose ester filters (Millipore) into acid-washed polyethylene Nalgene bottles to determine the concentrations of dissolved nitrogen (nitrate, nitrite, ammonium and urea), phosphate and silicate. Samples were frozen at −20°C until analyzed by Geochemical and Environmental Research Group, Texas A&M University. For the chl *a* samples, 400–800 mL seawater was filtered onto GF/F glass fiber filters (Whatman) then frozen immediately until analyzed by a calibrated Turner Designs model 10AU fluorometer. The extraction and calculation method were according to Quigg et al. [10].

At the beginning and the end of the bioassays, 1L-2L initial background water samples and 900 mL experimental water samples respectively were filtered onto GF/F glass fiber filters (Whatman). Filters were maintained in −80°C until reverse-phase HPLC analysis using the procedures of Pinckney et al. [31]. The HPLC instrument includes a binary gradient pump (Shimadzu dual LC10-ATvp and Controller SCL-10Avp), temperature controlled autosampler (Shimadzu SIL 10-Avp) with a 500 µL injection loop, column oven (Shimadzu CTO-10AS vp), and photodiode array detector (PDA, Shimadzu SPD-M10A vp; 200 to 800 nm). Pigments were extracted in 500–1000 µL cold 100% acetone with 100 µL synthetic carotenoid β-apo-8′-carotenal (internal standard) overnight. Before injection to the HPLC, samples were pre-filtered through a 0.2 µm PTFE (Gelman Acrodisc) filter. 300–400 µL extracted samples mixed with 1.0 mol L^−1^ ammonium acetate (ion-pairing solution) in a ratio of 4 (extracted sample):1(ammonium acetate) were added to the vials then placed in the autosampler rack for HPLC analysis. Pigments peaks were identified based on retention time and pigment spectra shape obtained from liquid standards (DHI, Hørsholm, Denmark).

Major phytoplankton groups were determined from the pigment compositions and by using the program CHEMTAX V1.95 (http://gcmd.nasa.gov/records/AADC_CHEMTAX.html). In our study, three different kinds of chlorophylls and 12 kinds of carotenoids were detected by HPLC analysis. The chlorophylls included chlorophyll *c* (chl *c*), chlorophyll *b* (chl *b*) and chlorophyll *a* (chl *a*), and the carotenoids included peridinin (peri), 19′-butfucoxanthin (but), fucoxanthin (fuco), 19′-hexfucoxanthin (hex), neoxanthin (neo), violaxanthin (vio), prasinoxanthin (pras), diadinoxanthin (diad), alloxanthin (allo), diatoxanthin (diat), lutein (lut), and zeaxanthin (zea). Eight groups of phytoplankton were defined from an earlier study in NGOM [32], but the pigment/chl *a* in the matrices were derived from multiple studies (see Table 1). Lewitus et al. [33] and Schlüter et al. [34] calculated the pigments ratios for a series lab-cultured costal phytoplankton species in different conditions, providing us reference to build the initial pigments ratio matrix in Chemtax, which was more suitable for costal studies than the matrix of Mackay et al. [35]. Based on the microscopic identification from selected samples we collected during the 2012 cruises, *Thalassiosira sp.* and *Prorocentrum sp.* were the most dominant species of diatoms and dinoflagellates, respectively, thus we applied the pigments ratios of *Thalassiosira minisoula* and *Prorocentrum minimum* from Lewitus et al. [33] to represent the two groups. The rest of the ratios were all the average values calculated from the pigment summary of Schlüter et al. [30] for multiple coastal species. For the microscopic identification, samples were preserved in 10% buffered formalin and identified to genus using Tomas [36].

**Table 1:** Initial pigment / chl *a* ratios for the different phytoplankton groups used for ChemTax V1.95.

Pigment Class	chl *c*	peri	but	fuco	hex	neo	vio	pras	diad	allo	diat	lut	zea	chl *b*	chl *a*
Diat	0.289	0	0	0.546	0	0	0	0	0.124	0	0.025	0	0	0	1
Dino	0.099	0.411	0	0	0	0	0	0	0.164	0	0.016	0	0	0	1
Cyan	0	0	0	0	0	0	0	0	0	0	0	0	1.245	0	1
Crypto	0.221	0	0	0	0	0	0	0	0	0.405	0	0	0	0	1
Prymn	0.137	0	0	0.031	0.625	0	0	0	0	0	0	0	0	0	1
Pelago	0.397	0	0.61	0.732	0	0	0	0	0.14	0	0.088	0	0	0	1
Prasino	0	0	0	0	0	0.096	0.069	0.229	0	0	0	0.067	0.051	0.605	1
Chloro	0	0	0	0	0	0.042	0.031	0	0	0	0	0.21	0	0	1

Diat  =  diatoms, Dino  =  dinoflagellates, Cyan  =  cyanobacteria, Crypto  =  cyptophytes, Prymn  =  prymnesiophytes, Pelago  =  pelagophytes, Prasino  =  prasinophytes, Chloro  =  chlorophytes. See text (methods) for pigment names.

### Data analysis

The statistical analysis was conducted using SPSS 13.0, and the figures were plotted using Matlab 7.11 or Sigmaplot 12.0. All data presented was calculated as means ± standard error (S.E.). One-way analysis of variance (one-way ANOVA) was used to determine the significance among different treatments, in which LSD test was used to group-paired significance test when the variance was homogeneous, and Dunnett's T3 test was applied to the heterogeneity of variance. Correlation test was used to test the correlation relationship between different parameters. *p*<0.05 was considered as significant.

## Results

### Hydrographic conditions and nutrients

The water column profiles prior to the start of each bioassay are shown in Figure 2, with surface and bottom values given in Table 2. Except for BA1, bioassays were started around noon. Photosynthetically active radiation (PAR) was highly variable reflecting sunny versus cloudy days at sea. The low PAR at the start of BA4 may have resulted in light limitation of phytoplankton productivity before incubation. The PAR in BA2 and BA3 represented normal conditions (sunny days) in April and August. PAR decreased with depth, but we still had detectable values in bottom waters in BA2 and BA3 (Fig. 2). Surface temperature was higher in August (31±1°C) than in April (23±1°C) (Table 2). There was no difference in temperature in the water column in April, while bottom waters were 3–5°C cooler in August (Fig. 2). Station A is closer to the Mississippi River plume which explains the lower surface salinity measured there than at station B which is located west of the Atchafalaya River (Figs. 1 and 2). Bottom water salinities were around 35±1 for all four bioassays (Table 2). In both April and August, DO in station A was higher than in station B (Fig. 2), consistent with the higher chl *a* values in station A (Table 2). The lowest DO values all appeared in the bottom. In BA3, bottom waters were hypoxic (<1.4 mL L^−1^ in [12]).

**Table 2:** Nutrients and hydrographic conditions at the bioassay stations immediately prior to starting the bioassays.

Variable	BA1sur	BA1bot	BA2sur	BA2bot	BA3sur	BA3bot	BA4sur	BA4bot
Nitrate + Nitrite (µmol L^−1^)	0.18	8.41	0.22	0.23	0.58	12.53	1.26	5.26
Ammonia (µmol L^−1^)	0.14	0.66	0.033	0.071	1.04	0.95	1.66	0.69
Phosphate (µmol L^−1^)	0.37	1.76	0.40	0.27	1.56	1.62	0.37	0.51
Silicate (µmol L^−1^)	0.37	18.9	4.24	5.04	31.32	40.36	6.97	26.80
DIN:Pi:Si	1∶1∶1	5∶1∶11	1.25∶1∶10.5	1.7∶1∶16.7	1.6∶1∶30	6.5∶1∶20	7.5∶1∶17.5	8.5∶1∶38.5
Chl a (µg L^−1^)	2.35	2.12	0.42	1.77	2.08	1.17	1.18	2.44
Temperature (^o^C)	23.2	23.0	23.1	23.8	31.9	26.6	30.8	28.2
Salinity	29.3	35.5	31.9	35.8	24.9	35.9	31.1	35.9
Incubation start time	8pm	8pm	1pm	1pm	2pm	2pm	2pm	2pm
Surface PAR (µmol quanta m^2^ s^−1^)	2.5	0	1875	115	1516	20.2	247	8.9

BA1 =  station A, April; BA2 =  station B, April; BA3 =  station A, August; and BA4 =  station B, August; sur  =  water collected in the top 2 m; bot  =  water collected between ∼15–18 m.

In NGOM, N, P, Si can all act as limiting nutrients for phytoplankton growth at times [10], [37]. The Redfield Ratio implies the average optimum nutrient ratio for phytoplankton is N:P:Si = 16∶1∶16. In NGOM, N and P limitation have been defined as DIN:P_i_<10 with DIN<1 µmol L^−1^ and DIN:P_i_>20 with P_i_<0.2 µmol L^−1^, respectively [10], [13]. In the lower Mississippi River, Si concentration has decreased and its ratio to N changed from 4∶1 in 1900s to 1∶1 in 1980s [37]. Based on this criterion, all our bioassays were conducted in N limited waters (Table 2). Si was more sufficient in August bioassays (BA3 and BA4), but BA1 phytoplankton were likely under Si limitation (Table 2). In BA1, BA3 and BA4, there were more nutrients (higher concentrations) at the bottom than the surface, indicating a potential nutrient pool for phytoplankton (Table 2).

### Response of phytoplankton biomass

**Figure 3:**
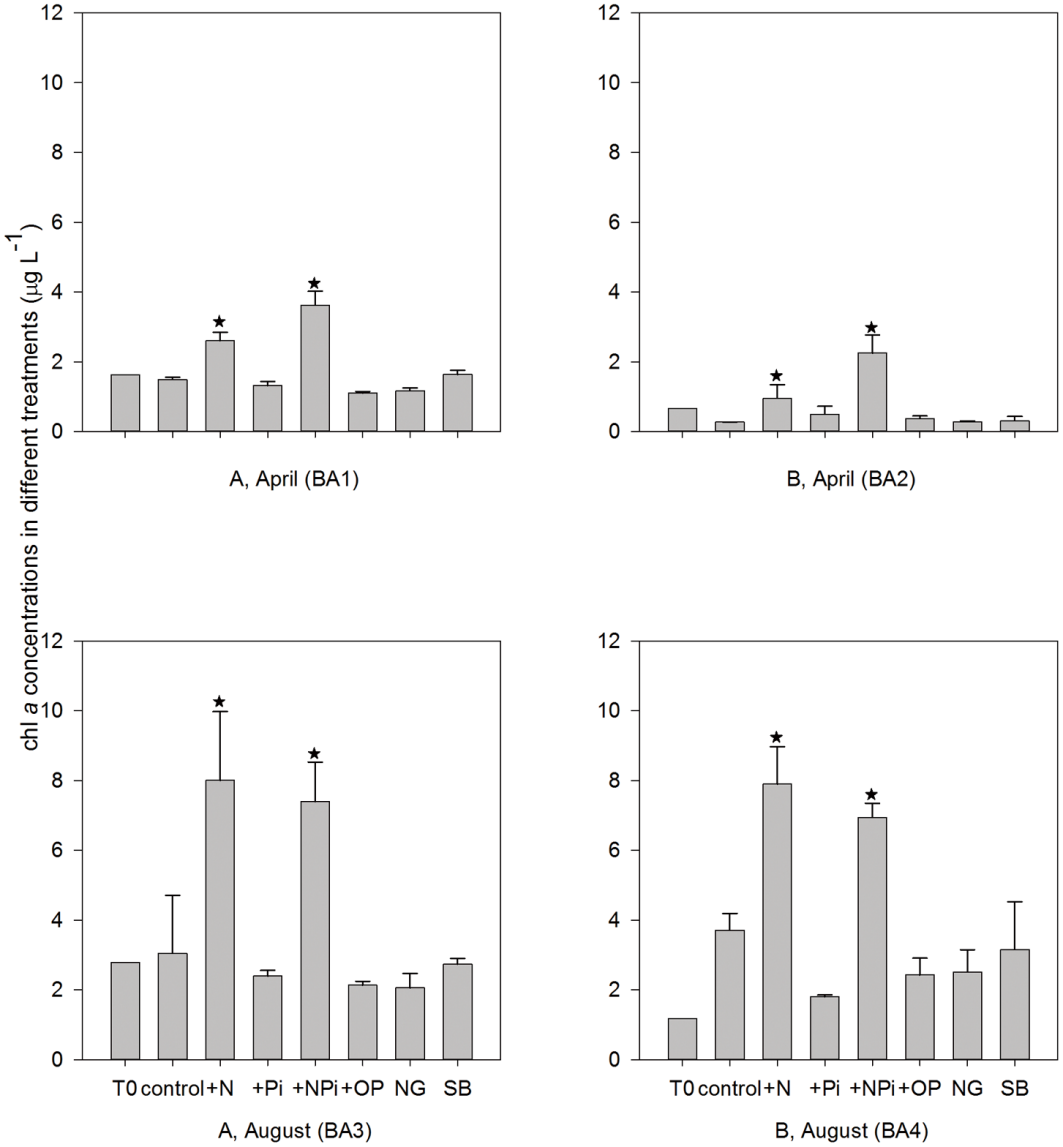
Chl *a* (µg L^−1^) concentrations in the different bioassay treatments (mean ± S.E). * indicates significant difference compared with the ‘control’ at the same time point. NG: no grazers, SB: surface+bottom. T_0_: chl *a* at start of the bioassay; control: chl *a* at the end of the bioassay (after 48 hours).

The change in chl *a* (µg L^−1^) concentrations in different treatments shared similar patterns in the four bioassays (Fig. 3). Only +N and +NP_i_ treatments showed significant stimulations at the end of the incubation (*p*<0.001, One-Way ANOVA). There was no significant difference among the other five treatments (*p*>0.05) relative to the T_0_ (chl *a* at start of the bioassay) and the control (chl *a* at the end of the bioassay). Compared to the T_0_, chl *a* concentrations in the control increased in BA4 (*p*<0.001). In BA1, BA2 and BA3, the chl *a* changes in controls were not statistically significant (Fig. 3). In April, chl *a* concentrations were higher in the +NP_i_ treatments than +N treatment (*p* = 0.012, 0.011, respectively). The situation was opposite in August but there was no statistically difference (*p* = 0.635, 0.221, respectively). The average chl *a* concentration increased 106% in April after N additions, but increased by 178% in August, indicating higher growth rates of phytoplankton in August than in April after nutrient additions. P additions (P_i_ and OP) and the removal of grazers did not result in an overall increase in chl *a* concentration in any bioassays compared to the control (Fig. 3). Similarly, the addition of ‘bottom’ waters to surface waters did not stimulate the growth of phytoplankton relative to the control treatments over 48 hours (Fig. 3).

**Figure 4:**
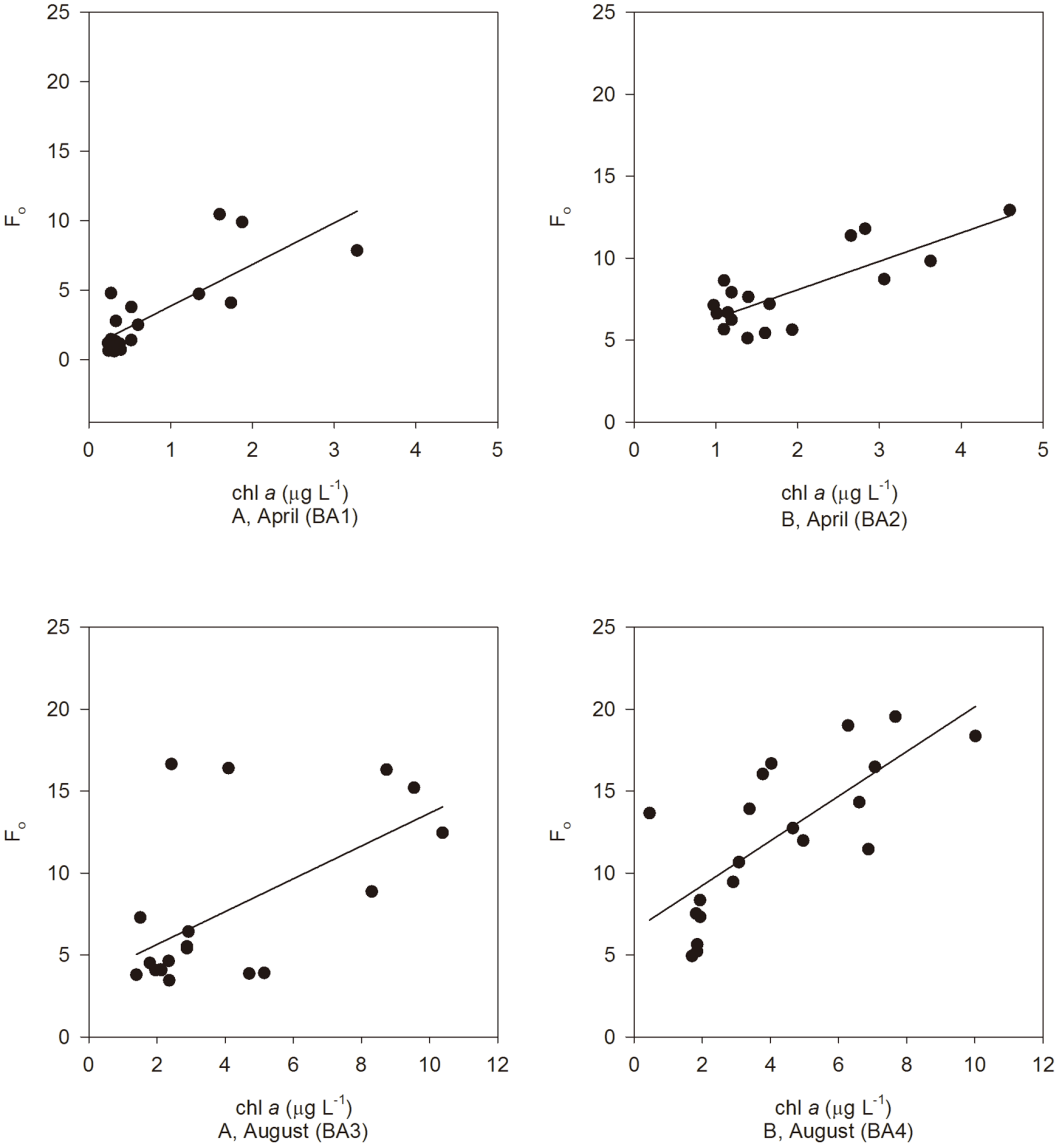
Linear relationship between F_o_ and chl *a* concentration (µg L^−1^) after 48 hrs incubation in different bioassays. R^2^ = 0.6216, *p*<0.001 in BA1, R^2^ = 0.6202, *p*<0.05 in BA2, R^2^ = 0.3503, *p*<0.01in BA3 and R^2^ = 0.5488, *p*<0.05 in BA4.

**Figure 5:**
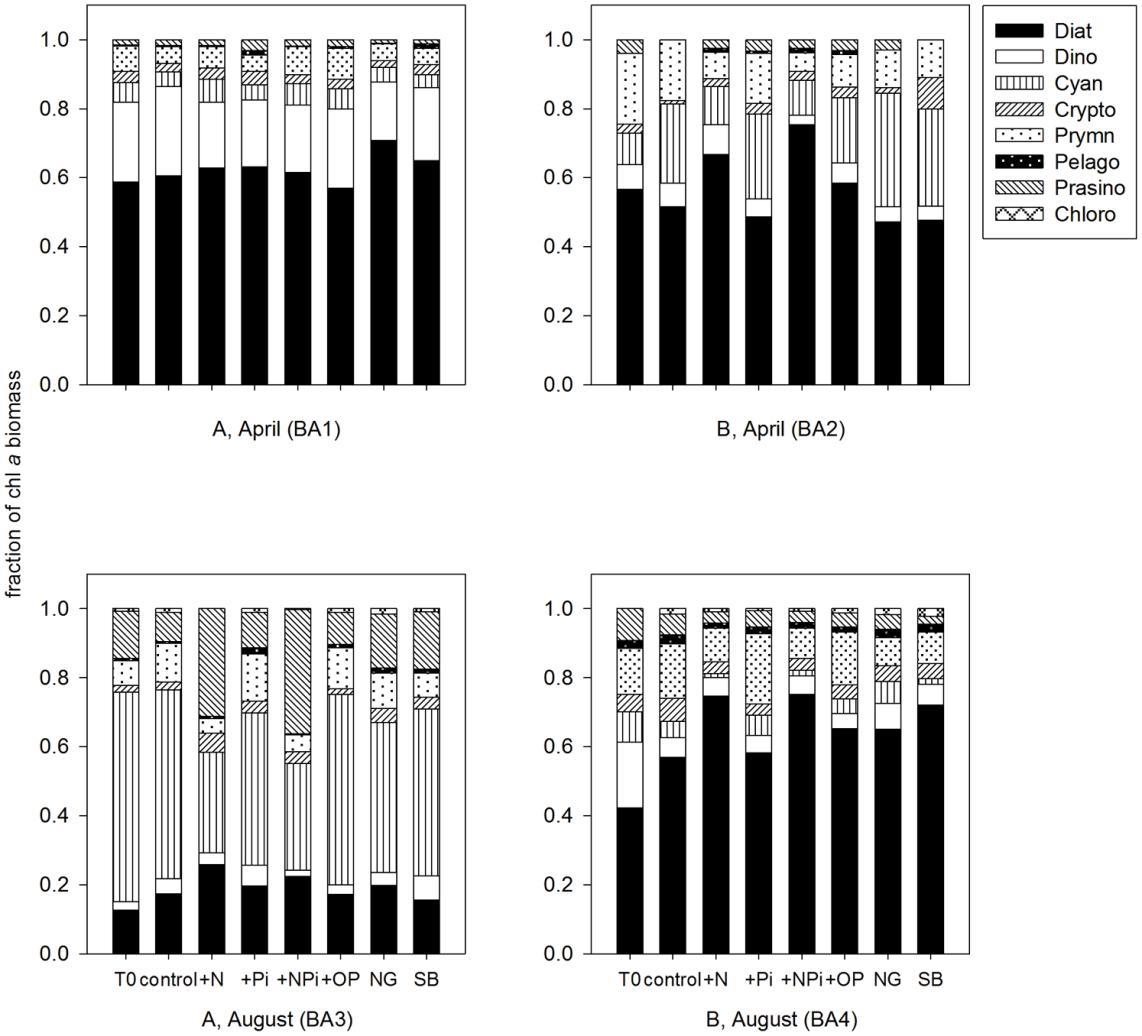
Phytoplankton community compositions in different treatments in the four bioassays at t = 48 hours determined by ChemTax V 1.95. NG: no grazers, SB: surface+bottom.

F_o_ is used to an estimate of the initial chl *a* fluorescence measured with the FIRe which can be used to represent chl *a* concentrations [27]. Given only small samples (3 mL) are required and the measurement is relatively fast, it can be used to provide greater temporal information than traditional measures of chl *a*. In our bioassays, F_o_ values were significantly correlated (linear relationships) to chl *a* concentrations (correlation analysis, *p*<0.001), regardless of station and cruise (Fig. 4). R^2^ value in BA3 equation was the lowest among the four bioassays, corresponding to the highest cyanobacterial abundance in BA3 among the four bioassays (Fig. 5). As with the Fast Repetition Rate fluorometer (FRRF), the PSII fluorescence yield of cyanobacteria containing phycocyanin instead of phycoerythrin cannot be efficiently harvested at the wave band of the instruments excitation [38]. Although the specific cyanobacterial taxa were not identified in our samples, earlier reports indicated taxa containing phycocyanin (eg. *Aphanizomenon* sp.) were common in our research areas [39].

**Figure 6:**
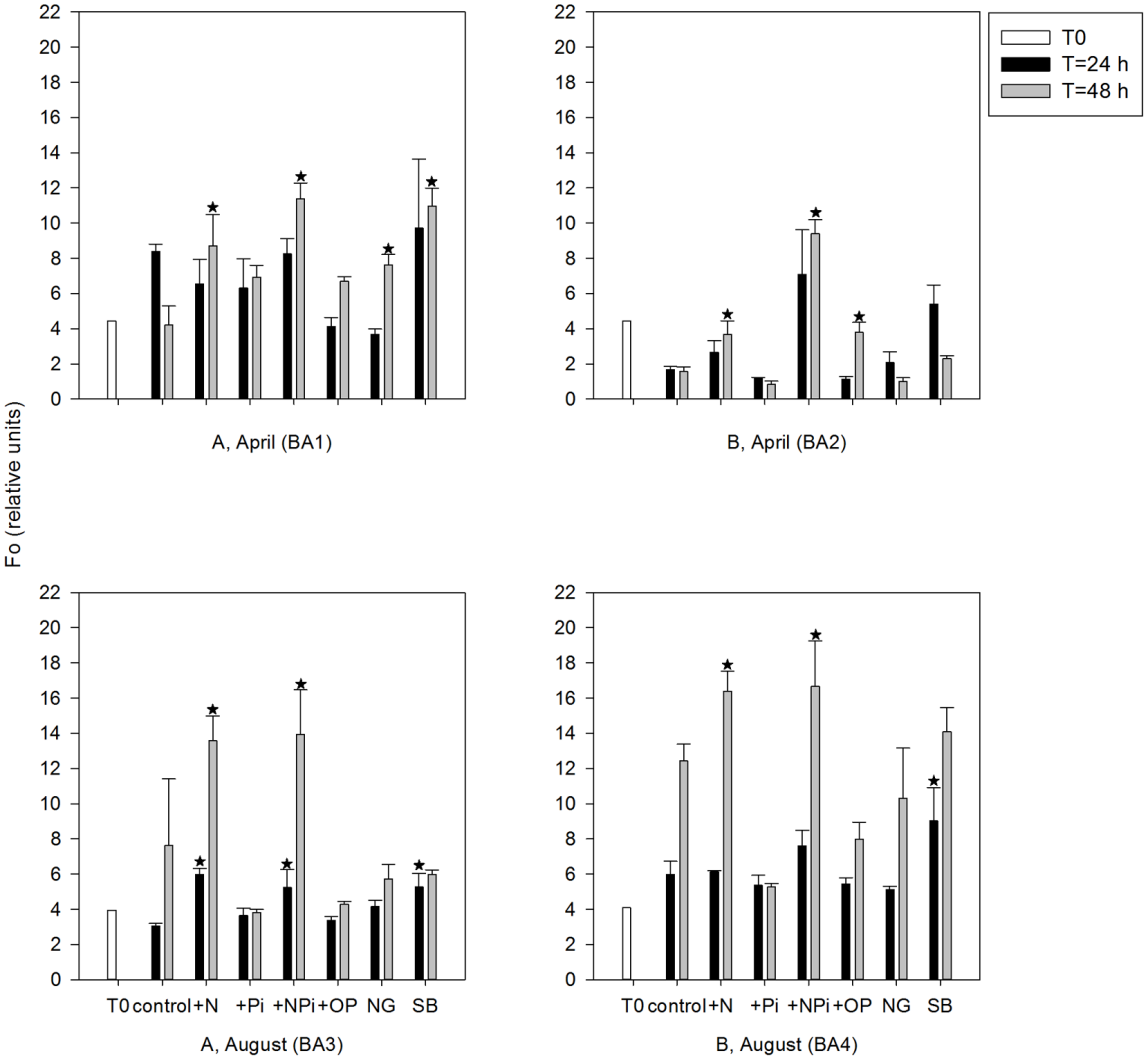
Variations in F_o_ values in measured in the different treatments. Data shown are the means ± S.E.. * indicates significant difference compared with the ‘control’ at the same time point. NG: no grazers, SB: surface+bottom. Unlike chl *a* which was measured only at the beginning (t = 0) and end (t = 48 hours), samples for fluorescence parameters were measured at t = 0, 24 and 48 hours.

At t = 48 hours, F_o_ values in N addition treatments showed the similar patterns with chl *a* changes, which were the significant stimulation effects after N and NP_i_ additions (*p*<0.001) (Fig. 6). There was significant F_o_ increase in the NG (no grazers) and SB (surface+bottom) treatments in BA1 and +OP treatments in BA2 (Fig. 6) (*p* = 0.036, 0.042, <0.001, respectively). Given we did not see the same pattern in the chl *a* data (Fig. 3), this maybe an overestimation of F_o_ by the FIRe. F_o_ in +N and +NP_i_ treatments started to show increase after the first 24 hours incubation in August bioassays (BA3) but not in April bioassays (*p* = 0.03, 0.045, respectively), showing a greater FIRe sensitivity in August (Fig. 6). In the first 24 hours, there was significant F_o_ increase in bottom water treatments in BA3 and BA4 (*p* = 0.003, 0.016, respectively), suggesting the short-term stimulated effects from bottom water, but this could not sustain phytoplankton to the end of incubations (Fig. 6).

### Response of photosynthetic activities

As Table 3 shown, F_v_/F_m_ values were not significantly different in the treatments (*p*>0.05) in April (BA1 and BA2) relative to the controls. In August (BA3 and BA4), F_v_/F_m_ values in the +N and +NP_i_ treatments were significantly higher than in other treatments (*p*<0.05), suggesting phytoplankton were recovering photosynthetic efficiency as a result of the alleviation from N limitation. Consistent with the chl *a* results, there were no effects of grazers, the addition of P (OP and P_i_) or bottom water on F_v_/F_m_ values in all the bioassays (Table 3).

**Table 3:** Variations in F_v_/F_m_ and *p* values derived from FIRe after 48 hrs of incubation.

	To	control	+N	+P_i_	+NP_i_	+OP	NG	SB
Fv/Fm								
A, April (BA1)	0.507(0)	0.43(0.027)	0.430(0.008)	0.437(0.008)	0.433(0.012)	0.417(0.032)	0.440(0.005)	0.407(0.035)
B, April (BA2)	0.382 (0)	0.546 (0.017)	0.557(0.007)	0.530(0.023)	0.531(0.038)	0.610(0.011)	0.543(0.018)	0.575(0.003)
A, August (BA3)	0.294(0)	0.343(0.026)	0.445(0.022)*	0.339(0.045)	0.434(0.004)*	0.327(0.026)	0.324(0.031)	0.296(0.026)
B, August (BA4)	0.521(0)	0.427(0.003)	0.507(0.008)*	0.437(0.008)	0.513(0.013)*	0.450(0.003)	0.490(0.023)	0.443(0.003)
*p*								
A, April (BA1)	0.06(0)	0.077(0.029)	0.11(0.006)	0.07(0.003)	0.135(0.043)	0.155(0.043)	0.07(0.005)	0.113(0.23)
B, April (BA2)	0.09 (0)	0.07(0.003)	0.07(0)	0.07(0.0033)	0.07(0)	0.07(0)	0.07(0)	0.07(0)
A, August (BA3)	0.05(0)	0.10(0.01)	0.14(0.015)	0.085(0.005)	0.10(0.003)	0.16(0.011)	0.11(0.008)	0.077(0.015)
B, August (BA4)	0.06(0)	0.107(0.018)	0.220(0.025)*	0.113(0.029)	0.200(0.04)*	0.120(0.021)	0.140(0.04)	0.130(0.036)

Data shown are the means ± S.E. * indicate significant difference compared with the control group.

There were three other photosynthetic parameters measured: σ_PSII_ (Å^2^ quanta^−1^), *p* (unitless) and τ_Qa_ (µs). These three parameters did not change in response to the treatments as was observed for F_o_ and F_v_/F_m_, with one exception. In BA4, *p* in +N and +NP_i_ treatments was significantly higher than the other groups (*p* = 0.045, 0.018, respectively) at the end of the incubation (Table 3). This was not the case in the other three bioassays (Table 3). The correlation between *p* and F_v_/F_m_ in BA4 was significant (correlation analysis, *p*<0.01). *p* is the connectivity factor; both N and P limitation could cause the decrease of *p* values [15]. The response of σ_PSII_ and τ_Qa_ to +N and +NP_i_ was not statistically different from the other treatments (*p* = 0.983, 0.972, respectively) and there was no consistent pattern among the four bioassays (Table 4).

**Table 4:** Average σPSII and τQA values measured in the four bioassays.

average	To	control	+N	+P_i_	+NP_i_	+OP	NG	SB
σ_PSII_ (Å^2^ quanta^−1^)	279(16)	373 (52)	398(48)	393(46)	425(58)	370(31)	378(26)	396(17)
τ_QA_ (µs)	977(166)	1007(128)	1251(350)	1096(276)	1276(375)	1274(341)	1143(238)	1127(278)

Data shown are the means ± S.E. * indicate significant difference compared with the control group.

### Response of phytoplankton communities

Chl *a*, fuco, hex, zea, chl *b*, and peri were the most abundant pigments in all the samples, indicating the dominant phytoplankton groups were likely to be diatoms, cyanobacteria, dinoflagellates, prymnesiophytes and prasinophytes (Fig. 5). These five groups accounted for more than 75% percentage of total phytoplankton biomass in all four bioassays. In all the bioassays, but, vio, allo and lut concentrations were in very low levels, representing low abundance of pelagophytes, cryptophytes and chlorophytes (Fig. 5). High chl *b* and pras concentrations in BA3 related to high abundance of green algae (prasinophytes) in only this bioassay.

Consistent with the chl *a* and F_o_ results, the most obvious shifts of phytoplankton community also happened in +N and +NP_i_ treatments (Fig. 5). Overall, there was a shift in phytoplankton communities from cyanobacteria and prymnesiophytes to diatoms and prasinophytes in BA2, BA3 and BA4 after N additions, while the community composition did not vary in different treatments in BA1. In BA2 and BA4, diatoms accounted for the highest percentage of the total phytoplankton compositions at the start (T_0_) and became more dominate after N additions at the end of the incubations. At the T_0_, the community compositions in BA3 were very different from the other three bioassays with cyanobacteria dominating and a high proportion of prasinophytes (Fig. 5). After N additions, the proportion of cyanobacteria almost equaled to diatoms and prasinophytes after 48 hours in BA3, because diatoms and prasinophytes were more stimulated by N additions than cyanobacteria. In BA1, although dinoflagellates accounted for more than 20% in the community compositions initially, their proportions did not show obvious changes after N additions (Fig. 5). For the other treatments, no shift happened to the phytoplankton compositions compared to the control group.

### Growth rates

The major groups of phytoplankton were stimulated by N additions to varying degrees. The growth rate (*μ*) for each group was calculated using 1/t (days)×ln(biomass _T = 48h_/biomass _T0_), in which biomass was estimated from “the absolute pigments compositions” calculated by ChemTax [40]. In the four bioassays, the average growth rates of the top five groups after N additions (the average values of +N, and +NP_i_ treatments) were diatoms > prasinophytes > dinoflagellates > prymnesiophytes > cyanobacteria, and the values were 0.718(±0.404) day^−1^, 0.565(±0.365) day^−1^, 0.343(±0.194) day^−1^, 0.301(±0.363) day^−1^ and 0.255 (±0.243) day^−1^, respectively. The large error associated with each growth rate reflects (1) seasonal difference (see Fig. 3) and (2) inherent differences between stations (see Fig. 1and 2).

## Discussion

The significant growth response of phytoplankton (F_o_ and chl *a*) to +N and +NP_i_ treatments and the nutrient data indicated the phytoplankton communities were N limited at the two experimental stations in both April and August 2012 in NGOM. N limitation usually occurs in mid-salinity areas (18–32), where station A and B were located [11]. N limitation has been reported in this area in both spring and summer [10], [41], and its effect on biological and chemical cycles in NGOM also has been emphasized in many studies (e.g. [7], [8], [11]). Reducing nitrogen load is considered as the key factor to reduce the phytoplankton biomass and alleviate the summer bottom hypoxia in NGOM [42]. N limitation of primary productivity has been reported in coastal ecosystems worldwide including the Baltic Sea [19], the Qatar peninsula in the Arabian Sea [25] and many other places as summarized in recent reviews by Howarth and Marino [43] and Paerl [44].

Si was plentiful for diatom growth except in BA1, performed in April adjacent to the Mississippi River station. Quigg et al. [10] also found evidence of Si limitation in March 2004 in the same area. We did not observe P limitation in all four bioassays, which was different from the former bioassays performed in NGOM [13], [15], [17]. P limitation was observed in March, May and July (spring and early summer) of 2004 [10] and with the surface salinity ranging from 10–35 (most happened between 10–20, [11]). The occurrence of P limitation resulted from the large amount of N loading from river inflow relative to P loading [17]; the river flow in 2012 was relatively low compared to 2010 and 2011 (http://www2.mvn.usace.army.mil/), which may explain the apparent absence of P limitation during our cruises.

The fast response (within 24 hours) of F_v_/F_m_ after the addition of nutrients in some but not all treatments is consistent with former studies (e.g. [15], [24]). If phytoplankton communities inhabit an oligotrophic environment for a long period and adapt to it (e.g., the North Atlantic Ocean), F_v_/F_m_ values will not change significantly after the relief of nutrient limitation; this is so called “balanced growth” [29], [30]. However, when the nutrient fluctuation frequency is high (e.g., NGOM), F_v_/F_m_ could respond more obviously to the additions of limited nutrients [15]. In our study area, the ambient nutrient conditions were more like the second situation although the F_v_/F_m_ changes in April (BA1 and BA2) were not obvious. More significant phytoplankton response after nutrient additions in summer time was also found by Mahaffey et al. [45]. The incubation effects generally indicated by the biomass decrease in BA2 (not statistically significant) and increase in BA4 (*p*<0.001) in controls could result from the further nutrient limitation in incubating bottles and initial light limitation in the two bioassays, respectively.

There were higher nutrient concentrations in the bottom waters than the surface waters in BA1, BA3 and BA4 (Table 2), which could stimulate phytoplankton communities. Although we did not observe significant bottom nutrient effects in our bioassays (see Fig. 6), it has been shown in other studies that the bottom water could act as the potential nutrient pool for phytoplankton in euphotic zones [45]. In NGOM, some extraordinary weather events, like the occurrence of hurricanes, could increase the possibilities of mixing bottom water to surface, adding nutrient pulse to stimulate phytoplankton bloom(s) [46]. Based on our study, small amounts of bottom water (10%) could not lead to changes in phytoplankton biomass and community structure in 48-hour bioassays, with some stimulation only in the first 24 hours.

High abundance of diatoms, cyanobacteria, dinoflagellates, prymnesiophytes and prasinophytes in our samples was consistent with former phytoplankton community studies in GOM [27], [47], [48]. The five phytoplankton groups were stimulated after N additions in the four bioassays. The shift of phytoplankton communities was the outcome of their different competitive abilities for N (nitrate in this case). According to trait-based approaches, we applied four nutrient- and group-specific functional traits (see Table 5): the maximum nutrient uptake rate (*V_max_*), the maximum growth rate (*μ_max_*), the minimum cell quota (*Q_min_*) and the half saturated constant (*K_s_*) to compare the nitrate competition ability among different phytoplankton groups and explain the community shifts after nitrate additions [20], [49].

**Table 5:** Different nitrate uptake related parameters in multiple marine species belonging to four eukaryotic phytoplankton groups, modified from Litchman et al. [20].

Phytoplankton groups	VmaxN (µmol N µmol C^−1^ day^−1^)	K_N_ (µmol)	µmax (day^−1^)	QminN (µmol N µmol C^−1^)
Diatoms	0.5–0.8	0.5–1.5	1.1–1.8	0.038–0.065
Green Alage	0.2	0.5–6	1.3–1.6	0.03
Dinoflagellates	∼0.0–0.1	2.5–6	0.3–0.7	0.015–0.035
Coccolithophores	0.06–0.08	0.2–0.5	1.1–1.2	0.02

In theory, *μ_max_* is more cell size related while *V_max_* and *K_s_* are more nutrient related [50]. *V_max_* for a specific kind of nutrients determines the performance of phytoplankton groups when this nutrient is sufficient in their habitats. The higher the *V_max_* is, the faster the phytoplankton group could take up the nutrient. *K_s_* represents the affinity for nutrients, and high affinity (low *K_s_*) for nutrients giving the phytoplankton group stronger competitive ability for nutrients in scarce environments [21], [51]. In our case, we focused on the response of different phytoplankton groups to nitrate additions in the N limited background environments, so *V_max_* for nitrate and *μ_max_* were more important to consider.

Litchman et al. [20] summarized multiple species values of *V_max_*, *K_s_*, *μ_max_* and *Q_min_* for nitrate competition (subset in Table 5). Due to the highest *V_maxN_* and *μ_max_* (small celled diatoms) and intracellular nitrate storage vacuoles (high *Q_min_*) in large diatoms, this group would show the strongest competitive abilities after nitrate additions (Table 5; [20], [22]). Prasinophytes (green algae) have the second highest *V_maxN_* and *μ_max_*, so they can take advantage in nitrate competition as well (Table 5; [20]). Prymnesiophytes take up nitrate slower than diatoms and prasinophytes, so they potentially are poor at competing for nitrate (Table 5; [20]). There was no information about cyanobacteria in Litchman et al. [20], but their ability for nitrate uptake should be the lowest among the five groups because of their smallest cell size [52]. For dinoflagellates, although their *V_maxN_* is higher than in prymnesiophytes and cyanobacteria, given they have a low *μ_max_*, they do not show high growth rates after nitrate additions. Based on the characteristics (four functional traits), the average growth rates after N additions should be diatoms> prasinophytes> dinoflagellates>prymnesiophytes >cyanobacteria, which is consistent with the patterns (Fig. 5) and the calculated growth rates in our bioassays.

**Figure 7:**
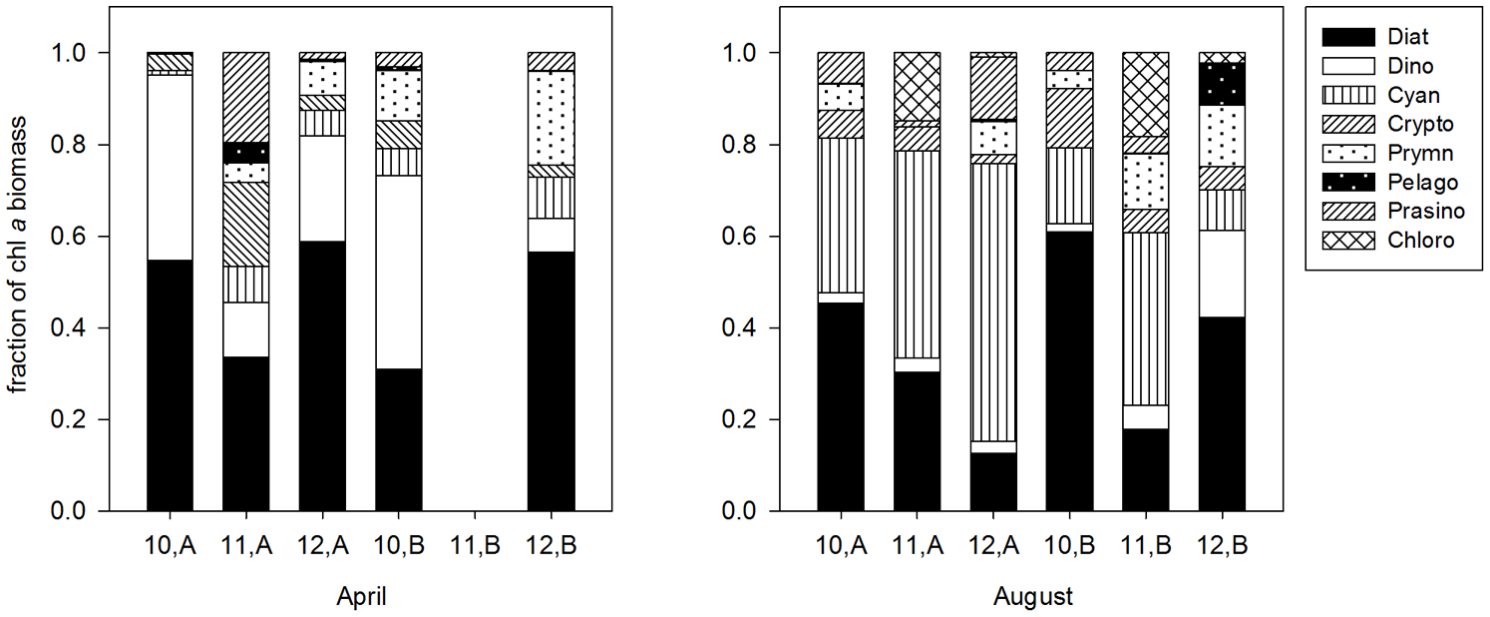
Phytoplankton community compositions in April and August 2010–2012, at station A and B. Data from surface samples around noon. Samples for 2011at station B in April were lost.

There are also biotic environmental factors that could influence the distribution of phytoplankton communities. For instance, diatoms are more adapted to low light, high turbulent environments, while prymnesiophytes favor sufficient light and calm water [20]. Cyanobacteria have the highest optimum growth temperature among the major five groups, which is why cyanobacteria usually dominate in summer [32], [50]. Green algae (prasinophytes, euglenophytes and chlorophytes) distribution is usually associated with low salinity or estuary water [53]. Based on our historical data at the same stations (A, B) from 2010 to 2012 cruise (Fig. 7), the average phytoplankton community composition was diatoms (or dinoflagellates) dominating in spring and cyanobacteria dominating in summer. The seasonal shift from large-celled diatoms (or green algae in freshwater systems) blooms to small-celled cyanobacteria blooms was the typical situation in both marine [32], [54] and freshwater systems [55], [56]. At station B, August, in 2010 and 2012, diatoms dominated over cyanobacteria, which might be a temporal phenomenon related to windy and rainy weather during the cruise, because coastal diatoms could take more advantages in the fluctuating light conditions [57].

The responses of phytoplankton communities in the four bioassays support the applicability of trait-based ecological approaches to evaluate short-term changes in phytoplankton community structure in the field [20], [22]. Long-term RLAs conducted in other marine ecosystems also supported our short-term results. For example, Lugus et al. [19] found centric diatoms were the most stimulated phytoplankton group and a big decline of autotrophic picoplankton in 14-day RLAs in N limited northern Baltic Sea. The response of dinoflagellates was highly species-specific in the Baltic study; this supported our finding of variability in the pattern of dinoflagellates responses. Mahaffey et al. [45] mixed nutrient-enriched bottom water with oligotrophic surface water in the North Pacific Subtropical Gyre and within 5 days, found similar community shifts including a change from small cells to large cells as we hypothesized. We did not find obvious effect of grazers in all the bioassays, consistent with Lugus et al. [19], thus the response of the phytoplankton communities after N addition was mainly controlled by bottom-up effects, not top-down effects.

Because picoplankton have slow sedimentation rates and tight grazing effects by microzooplankton, most of them can be recycled in euphotic zone [58]. Microzooplankton (<20 µm, protozoa) dominated in our research area (mid-salinity 18–32) and peaked in summer [9]; this is the major grazer for picoplankton. It is hypothesized that increased phytoplankton biomass could induce more severe hypoxia in NGOM, but not many studies examined the outcome of community shifts [12]. In NGOM, the decomposition of diatoms contributed to large proportion of the bottom oxygen consuming, especially in spring when zooplankton biomass has not peaked [9], [16]. Additionally, diatoms are the major food source of zooplankton, and the fecal pellets produced by zooplankton also could sink to the bottom acting as oxygen consuming organic matters [9]. For the large contribution of sinking particles, diatoms were considered as an important trigger for the bottom hypoxia when the other involved hydrographic conditions are suitable [12], [16]. Therefore, the increased proportion of diatoms could result in more sinking diatoms, more zooplankton fecal pellets, thus more hypoxia potential. Dortch and Whitledge [16] proposed another scenario that Si limitation but sufficient N, P could cause the shift from diatom to some noxious flagellates, which was the situation at station A, April. Based on model assimilations, Eldridge and Roelke [59] also indicated that less edible species dominated in phytoplankton assemblages could enhance the potential of hypoxia, even their growth rates were lower than the edible species. Under this scenario, decreased grazing rates for dinoflagellates will also result in more sinking cells although the growth rates of dinoflagellates are not as high as diatoms.

To conclude, our research results indicated the FIRe could be used for detecting N limitation in NGOM, in which F_o_ and F_v_/F_m_ performed better than the other fluorescence parameters. The response of phytoplankton communities corresponded to the classic nutrients competition theories, providing evidence for the applicability of trait-based ecology in coastal phytoplankton communities. Furthermore, the dominating phytoplankton group shifted to diatoms (like in BA3) after nitrate addition reflected the shift trend from small-celled phytoplankton groups to large-celled ones when more ambient nutrients were available. Phytoplankton cells are considered as a major component of particulate flux in empirical and model calculations [60]. Therefore, the size shift of sinking phytoplankton cells could lead to a more complex impact on ecosystem in NGOM than merely considering total phytoplankton biomass.
